# Infants’ Implicit Rhyme Perception in Child Songs and Its Relationship With Vocabulary

**DOI:** 10.3389/fpsyg.2021.680882

**Published:** 2021-09-06

**Authors:** Laura E. Hahn, Titia Benders, Paula Fikkert, Tineke M. Snijders

**Affiliations:** ^1^Centre for Language Studies, Radboud University Nijmegen, Nijmegen, Netherlands; ^2^International Max Planck Research School for the Language Sciences, Nijmegen, Netherlands; ^3^Department of Linguistics, Faculty of Human Sciences, Macquarie University, Sydney, NSW, Australia; ^4^Max Planck Institute for Psycholinguistics, Nijmegen, Netherlands; ^5^Donders Institute for Brain, Cognition and Behaviour, Radboud University Nijmegen, Nijmegen, Netherlands

**Keywords:** rhyme, songs, vocabulary, ERP, infant

## Abstract

Rhyme perception is an important predictor for future literacy. Assessing rhyme abilities, however, commonly requires children to make explicit rhyme judgements on single words. Here we explored whether infants already implicitly process rhymes in natural rhyming contexts (child songs) and whether this response correlates with later vocabulary size. In a passive listening ERP study, 10.5 month-old Dutch infants were exposed to rhyming and non-rhyming child songs. Two types of rhyme effects were analysed: (1) ERPs elicited by the first rhyme occurring in each song (rhyme sensitivity) and (2) ERPs elicited by rhymes repeating after the first rhyme in each song (rhyme repetition). Only for the latter a tentative negativity for rhymes from 0 to 200 ms after the onset of the rhyme word was found. This rhyme repetition effect correlated with productive vocabulary at 18 months-old, but not with any other vocabulary measure (perception at 10.5 or 18 months-old). While awaiting future replication, the study indicates precursors of phonological awareness already during infancy and with ecologically valid linguistic stimuli.

## Introduction

Being able to judge whether words rhyme is one of the earliest forms of phonological awareness children develop (Ziegler and Goswami, 2005). Rhyme awareness reflects children’s metalinguistic ability to separate the initial phoneme of a syllable from the rest and is usually assessed with a rhyme judgement task, in which children decide whether a set of words rhymes (e.g., *king/ring*) or not (e.g., *king/pear*). Measured from around 4 years of age (Bryant and Bradley, 1978; Bradley and Bryant, 1983), rhyme awareness, among other phonological awareness measures, serves as a standard predictor for future literacy (Wood and Terrell, 1998; Melby-Lervåg et al., 2012).

Rhymes already play a role in the developing lexicons of much younger children. Many frequent words in children’s input rhyme (e.g., *hit, bit, pit*) (De Cara and Goswami, 2002), presumably resulting in the first words in infants lexicons to also rhyme, rather than to share phonemes at the word onset (e.g., *pin/bin* vs. *pin/pit*, respectively) (Zamuner, 2009). It has been suggested that the necessity to differentiate and recognise such similar-sounding words causes children’s initially holistic lexical representations to be re-structured into segmental-level specifications (Metsala and Walley, 1998; Fikkert and Levelt, 2008). Consequently, a growing lexicon might lead to an increase in phonological awareness (Metsala and Walley, 1998; Carroll et al., 2003) and this relationship is probably reciprocal: infants’ developing phonological sensitivity also enables a further growth in the number of lexical representations (Curtin and Zamuner, 2014). In fact, there is a well-established association between pre-school phonological awareness and vocabulary size (e.g., Lonigan et al., 2000; Stadler et al., 2007), where phonological awareness is mainly assessed with explicit rhyme judgement tasks and has been attributed to a need to differentiate words with offset-overlap in growing lexicons (Metsala and Walley, 1998; De Cara and Goswami, 2002, 2003).

Despite the evidence regarding the role of rhyming words in infants’ lexicons, previous studies have been equivocal concerning infants’ ability to recognise rhymes. This is mainly due to differences in test procedures and stimuli: while infants respond to a change from one single-word rhyme pattern to another in the Conditioned-Headturn-Procedure (Hayes et al., 2000, 2009), 9-month-olds may only display spontaneous differentiation between rhyming and non-rhyming child songs (Hahn et al., 2018), but not between rhyming and non-rhyming word lists (Jusczyk et al., 1999). The tentative evidence in favour of infants’ rhyme detection in Hayes et al. (2000, 2009) and Hahn et al. (2018), suggests that infants’ rhyme detection might be subtle. An implicit paradigm, like passive-listening EEG, might therefore be more sensitive to infants’ emerging rhyme abilities (Kooijman et al., 2008).

The results by Hahn et al. (2018) suggest that infants are able to recognise recurring rhymes in their natural linguistic context. Caregivers rhyme and sing for their infants on a daily basis (Ilari, 2005) and it has been suggested that at-home musical and language play contributes to vocabulary growth (Franco et al., 2021) and emergent literacy (Politimou et al., 2019; Krijnen et al., 2020). Specifically, toddlers’ experience with nursery rhymes was associated with several phonological abilities in a number of studies (Bryant and Bradley, 1978; Bradley and Bryant, 1983; Dunst et al., 2011) and caregiver singing during infancy positively influences later vocabulary size (Franco et al., 2021). Potentially, the acoustic shape of songs creates a perceptual boost for infants, due to rhymes in songs being placed at a salient phrase-final position and in a predictable rhythmic context (Kotz and Schwartze, 2010). In the current study, we employ a passive-listening EEG paradigm to answer the following research questions: (1) Do infants process rhyming songs differently from non-rhyming songs? and (2) Is the ability to detect recurring rhymes in songs related to later vocabulary?

The ERP literature on rhyme processing contains a range of effects, including the classic N450 rhyme effect, a negativity for non-rhymes at posterior electrodes elicited during explicit rhyme judgement tasks (Rugg, 1984a,b). There is no consensus whether pre-literate children already show this effect (Wagensveld et al., 2013; Andersson et al., 2018), but one study observing the N450 at this young age found that it correlated with phonological awareness (Andersson et al., 2018). An anterior negativity for rhyming pseudo-words has been reported for 4-year-olds in the absence of a rhyme task (Andersson et al., 2018), suggesting that the anterior negativity reflects implicit automatic rhyme processing. Note, however, that pre-schoolers executing rhyme judgements also displayed an early anterior negativity, which reduced in amplitude with increased letter knowledge (Wagensveld et al., 2013). In the present study, we expect an early negativity for rhymes, most likely at anterior electrode sites, as the infants will not be executing a task and still have limited phonological awareness.

The infant (EEG) research tradition has not yet assessed infants’ rhyme abilities, but laid the foundation by providing evidence for infants’ ability to detect repeated phonemes and words in speech and associating this detection with vocabulary size (see Cristia et al., 2014 for review). Specifically, the ERP word familiarity effect usually occurs between 200 and 500 ms after word onset as a left anterior negativity for the familiar word, which becomes more negative with each repetition. The effect occurs in response to several repetitions (e.g., Kooijman et al., 2005), but also after a single repetition of the same word (e.g., Kidd et al., 2018) in continuous speech, and is influenced by stimulus features and task difficulty (Mills et al., 2004; Junge et al., 2012; Snijders et al., 2020). For example, Snijders et al. (2020) observed a positive word familiarity effect for words occurring in child songs. The word familiarity effect has been established as a reflection of infants’ ability to recognise repeating words in speech (see e.g., Teixidó et al., 2018 for review). Individual differences in the polarity of the ERP word familiarity effect are associated with vocabulary size (Kooijman et al., 2013; Junge and Cutler, 2014; Kidd et al., 2018): Infants with more negative word familiarity effects tend to have larger vocabularies.

The current study builds on the discussed EEG research on rhyme processing with adults and children in combination with the infant word familiarity effect to ask whether infants detect rhymes in songs and whether individual differences in this ability are related to infant vocabularies. We specifically aim to extend the word familiarity effect to another phonological unit: rhymes. We presented 10.5-month-old Dutch infants with child songs of 10 phrases long from Hahn et al. (2018) in a rhyming and non-rhyming version, which only differed in the final pseudo-word at the end of every phrase (e.g., *paf, taf, kaf* vs. *teet, deus, bag*).

Two types of effect will be investigated*: rhyme sensitivity* and *rhyme repetition*. Rhyme sensitivity will be measured at the first point of diversion between rhyming and non-rhyming songs, i.e., at the end of the second phrase where the rhyme is repeated for the first time in rhyming songs but not repeated in non-rhyming songs. Measuring rhyme sensitivity corresponds to earlier studies where critical words (Junge et al., 2012; Kidd et al., 2018) or rhymes (Andersson et al., 2018) were repeated only once. The rhyme repetition effect will be measured as the averaged response to rhymes occurring at the end of the 3rd through 10th phrase of the songs. Measuring responses to repeated rhymes is comparable to the ERP word familiarity response to words repeated across successive sentences (Kooijman et al., 2005; Junge et al., 2012). For both effects, we expect a left anterior negativity for rhymes, which might be more pronounced in the rhyme repetition effect due to repetition enhancement (Nordt et al., 2016). The onset of the ERP effects is probably slightly later than the 200 ms reported in earlier word familiarity studies (Teixidó et al., 2018), due to the beginning of the phonological overlap being shifted to after the onset phoneme. Finally, both ERP effects will be correlated with Dutch CDI scores (Zink and Lejaegere, 2002) for productive and receptive vocabulary at 10.5 and 18 months, to investigate a possible link between rhyme perception and vocabulary size.

## Methods

This study was approved by the local ethics committee and parents of participating infants gave informed consent prior to data collection in accordance with the Declaration of Helsinki.

### Participants

In total 40 10.5-month-old infants from monolingual Dutch households were tested, from which 12 infants were excluded from data analysis due to not contributing at least 30 trials each for rhyming and non-rhyming songs (5 infants) or due to having more than two neighbouring noisy channels (7 infants). Twenty-eight datasets were used for the rhyme repetition effect (see section “Statistical analysis” below): mean age: 320 days, range: 304–338, 13 girls. From this subsample, 18 infants contributed enough trials to be analysed for the rhyme sensitivity effect (see section “Statistical analysis” section below). Sample size was determined prior to data collection based on similar studies by Kooijman et al. (2005, 2013) and Junge et al. (2012, 2014).

### Stimuli

**FIGURE 1:**
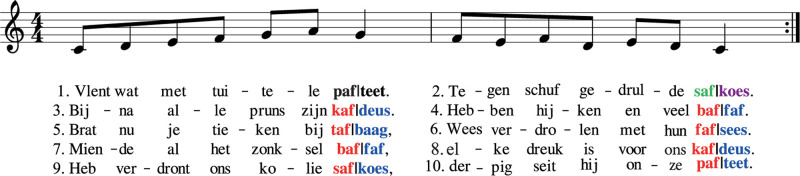
Example Stimulus. Song with lyrics in non-sense Dutch. Critical pseudo-words used to assess the rhyme sensitivity effect at the end of phrase 2 of the songs highlighted in green (rhyme) and purple (non-rhyme); Critical pseudo-words to assess the rhyme repetition effect from phrase 3 to 10 of the songs are highlighted in red (rhyme) and blue (non-rhyme). Stimuli were taken from Hahn et al. (2018). For the analysis, the first phrase-final pseudo-word of every song (in bold) was disregarded, as this cannot rhyme.

Song stimuli were taken from Hahn et al. (2018) and comprised of nine novel songs that were unknown to the infants. An example stimulus is depicted in Figure 1. See the original publication for more detail on creation and acoustic characteristics of the songs and a link to the song stimuli.

Each song occurred in two versions (rhyming and non-rhyming) and consisted of ten phrases (five verses consisting of two phrases each). Song lyrics were in non-sense Dutch to control for word familiarity: content words were replaced with legal Dutch pseudo-words, while function words were regular Dutch function words. Critical words at the end of every phrase comprised of CVC pseudo-words. Rimes of CVC pseudo-words were of medium frequency and all occurred as word ends in the Dutch Celex (Baayen et al., 1995), while the full CVC pseudo-words (including the initial consonant) did not appear in Celex or had a very low frequency (<100 raw). The full songs were on average 33 s long (range rhyming: 28–36 s, range non-rhyming: 28–35 s), including 500 ms between consecutive phrases of every song. The ten single phrases of each song were on average 2.7 s long (range rhyming: 2.2–3.3 s, range non-rhyming: 2.2–3.2 s). Critical pseudo-words were on average 690 ms long (ranges rhyming: 511–1022 ms, range non-rhyming: 489–908 ms). The rhyming and non-rhyming version of a song were identical apart from the final pseudo-word of every phrase (critical pseudo-word). These either rhymed (e.g., *paf, taf, kaf*) for the rhyming version of a song, or did not rhyme (e.g., *teet, deus, bag*) for the non-rhyming version.

### Procedure

Each test session was run by two experimenters. One experimenter briefed the parents and ran the measurement, while the other entertained the infant during placement of the electrode cap and data collection. The entire testing procedure from arrival to farewell lasted approximately 1 h. During the test session, the infant sat on a caregiver’s lab in an electrically shielded room. Silent baby-friendly movie clips were played at a PC screen in front of the infant. One experimenter sat next to the caregiver to silently entertain the infant during the measurement if necessary. Both caregiver and experimenter listened to masking music over headphones throughout data collection.

Stimulus presentation was controlled by Presentation software (Neurobehavioral Systems, Inc., Berkeley, CA^1^). The nine song stimuli (ten phrases each) in their two versions (rhyming/non-rhyming) were randomised across two experimental blocks. Songs of the same condition (rhyming/non-rhyming) never occurred more than twice in a row. Data collection lasted around 11 min.

Parents filled in questionnaires on their musical and demographic background and the vocabulary of their child (N-CDI 1 at 10.5 and N-CDI 2B at 18 months of age). So far, only the vocabulary questionnaires have been analysed.

EEG activity was collected from 32 Ag/AgCl electrodes (ActiCAP) using BrainAmp DC and BrainVision Recorder Software (Brain Products GmbH, Germany). Electrode locations were in accordance with an extended 10/20 system: F7/3/4/8, FC5/1/2/6, C3/4, CP5/1/2/6, P7/3/4/8, Fz, FCz, Cz, CPz, Pz, POz for collection of EEG activity. Electro-oculogram (EOG) was recorded using an electrode on the left or right cheek and above the eye (Fp1/2) for vertical EOG, and left and right of the eyes (FT9/10) for horizontal EOG. AFz served as Ground, FCz as online reference. Impedance were typically kept below 25 kΩ. Data was collected with a sampling rate of 500 Hz using an online low-cut off filter of 10 s and high-cut off of 1000 Hz.

### Data Preprocessing

EEG data were analysed in MATLAB (The MathWorks, Natick, MA, United States) using the Fieldtrip toolbox (Oostenveld et al., 2011). Data was filtered offline at 0.1–30 Hz. For one infant, a 0.5 Hz high-pass filter was used, due to slow drifts during the measurement. Bad channels were manually removed, as were data segments with flat channels or large artefacts (>150 μV for EEG channels, >250 μV for EOG channels). Eye- and single-electrode noise components were identified using Independent Component Analysis (Makeig et al., 1996) as implemented in the EEGlab toolbox (Delorme and Makeig, 2004) with infomax ICA (Bell and Sejnowski, 1995) on 1-s data snippets.

Critical pseudo-words were the final pseudo-words from phrase 2 to 10 from every song, resulting in 162 possible trials in total (see Figure 1 for an example, the rhyming (green/red) and non-rhyming (purple/blue) pseudo-words are the critical pseudo-words). Raw data was epoched from −200 to 900 ms around critical pseudo-word onset, using 0.1–30 Hz filters (0.5 Hz high-pass filter for one infant), and the eye- and noise-components identified with ICA were removed from the data (average of two eye and three noise components per infant). Critical pseudo-word-epochs were re-referenced to linked-mastoids. For three infants a single mastoid electrode was used as a reference, due to the other reference electrode being noisy. Time-locked data was baseline corrected by normalising waveforms relative to the 200 ms epoch preceding the onset of the critical pseudo-word. Trials containing activity exceeding ±150 μV were removed, leading to exclusion of five infants who did not contribute a minimum of 30 of the 81 possible trials for rhyming and non-rhyming songs. Nine channels were not analysed due to being noisy in too many datasets: F7/F8, F3/F2, Fz, T7/8, P7/8. Further noisy/missing channels were repaired using spline interpolation and a custom neighbourhood structure (a total of 17 channels repaired in 16 infants). Seven infants were excluded from further data analysis due to having more than two neighbouring noisy channels, making channel repair unreliable. Event-related potentials were computed for the remaining 13 channels (FC5/6, FCz, C3/4, Cz, CP5/6, CP1/2, P3/4, Pz) by averaging the rhyming and non-rhyming trials.

### Statistical Analysis

Two ERP effects were investigated: (1) the *rhyme sensitivity effect*, only on ERPs from phrase 2 of the songs, the moment where rhyming and non-rhyming songs first differed (green/purple in example Figure 1) (minimum of five trials per condition for this analysis, *Mean (SD)* number of trials rhyming: 7 (*1.09*), and non-rhyming trials: 7 (*1.47*), *n* = 18 infants) and (2) the *rhyme repetition effect*, averaged over ERPs from phrase 3 to 10 of every song (red/blue in example Figure 1, *n* = 28 infants, *Mean (SD)* number of trials rhyming: 48 (*11.43*), and non-rhyming trials: 48 (*10.39*)). Non-parametric cluster-based permutation tests (Maris and Oostenveld, 2007) were used to evaluate differences in the ERPs between the rhyming and non-rhyming conditions. For these tests, first dependent-samples t-tests are calculated to compare rhyming and non-rhyming conditions (for all 13 remaining electrodes and all time-points between 0 and 900 ms after onset of the critical pseudo-word). Then, clusters are made of neighbouring electrodes and time-points that exceed a threshold alpha of 0.05 (uncorrected). A cluster-level statistic (sum of t-statistics in the cluster) is then computed and, using Monte-Carlo resampling (1000 permutations), a reference distribution is made for random data, to which the observed cluster-statistic is compared to get a Monte Carlo *p*-value. This effectively controls for multiple comparisons while taking the electrophysiological properties of EEG into account (Maris and Oostenveld, 2007; Luck and Gaspelin, 2017; Sassenhagen and Draschkow, 2019).

From the N-CDI questionnaire for each infant the following scores were derived: comprehension at 10.5 months of age and production and comprehension at 18 months of age. To adhere to previous research (e.g., Kidd et al., 2018), non-parametric Kendall’s Tau rank correlations were calculated to investigate the relationship between ERPs (mean of cluster electrodes and cluster time-points of identified clusters in cluster-based permutation test comparing rhyming and non-rhyming conditions) and the vocabulary scores (not normally distributed).

## Results

### Rhyme Sensitivity Effect

**FIGURE 2:**
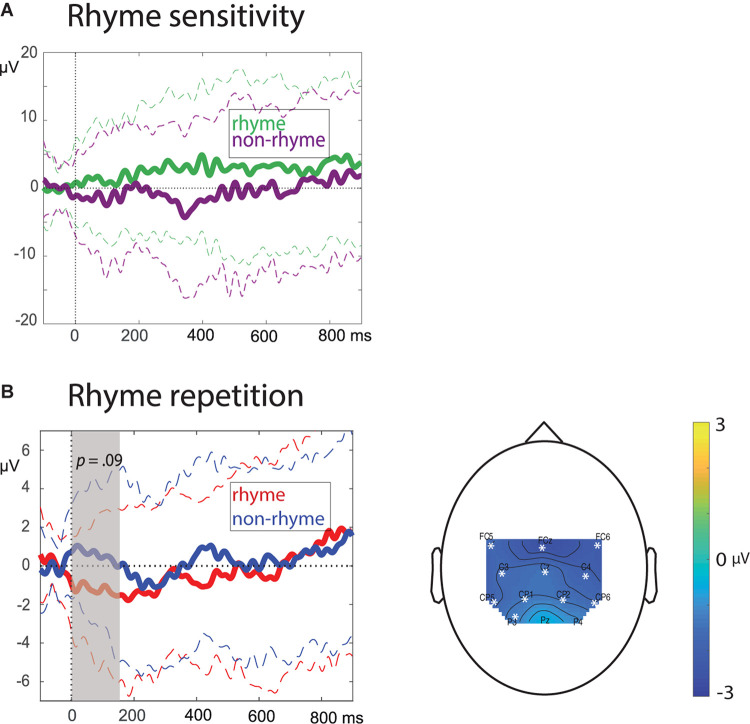
Rhyme sensitivity effect (based on the final pseudo-word of phrase 2 of every song, *n* = 18 infants) and Rhyme repetition effect (based on the final pseudo-words of phrase 3–10 of the songs, *n* = 28 infants). Solid lines = Means; dotted lines = ±1 SD; **(A)**: ERPs, averaged over all 13 electrodes; **(B)** left: ERPs averaged over the electrodes in the largest identified cluster (0–178 ms, *p* = 0.09), right: topographic isovoltage maps of the difference between rhyme and non-rhyme within the rhyme repetition effect cluster time window (0–178 ms) for all tested electrodes, cluster electrodes are marked with white star.

In response to the first occurrence of the rhyme/non-rhyme (phrase 2 of each song), rhyming pseudo-words induced a more positive ERP waveform compared to non-rhyming pseudo-words (Figure 2A). This difference was not significant (lowest cluster *p* = 0.6).

### Rhyme Repetition Effect

ERPs for rhyming pseudo-words occurring at the end of phrase 3–10 of the songs were more negative than ERPs for non-rhyming pseudo-words within the first 200 ms after pseudo-word onset (Figure 2B). None of the identified clusters in the cluster-randomisation test of the 0–900 ms time-window survived multiple comparisons correction (lowest cluster *p* = *0.09*). The cluster with the lowest *p*-value ranged from 0 to 178 ms and contained all electrodes except for Pz and P4: *Mean (SD)* rhyme: −1.27 (2.55), *Mean (SD)* non-rhyme = 0.48 (2.57).

**FIGURE 3:**
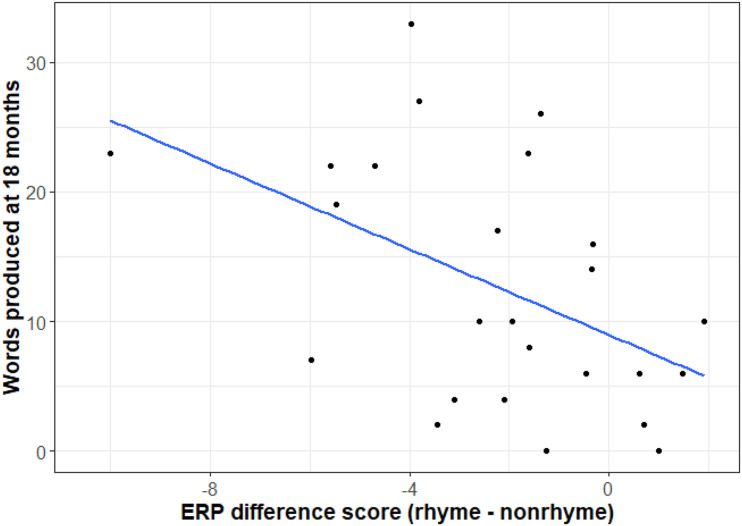
Correlation between words produced at 18 months (y-axis) and ERP difference scores (rhyme – non-rhyme, averaged for the cluster electrodes and time window) on the x-axis: τ = -0.3, *p* = 0.03. The correlation remains marginally significant when excluding the outlier at (−10, 23): τ = -0.3, *p* = 0.07.

Individual differences in the *rhyme repetition effect* within the largest identified cluster (mean of all cluster electrodes within the 0–178 ms time window) were significantly correlated with productive vocabulary at 18 months (τ = -0.3, *p* = 0.03). Infants with a larger negative ERP difference (rhyme more negative than non-rhyme) produced more words at 18 months old (Figure 3). There were no correlations with comprehension at 10.5 or 18 months.

## Discussion

Explicit phonological awareness during preschool years is an important predictor for literacy (Wood and Terrell, 1998; Melby-Lervåg et al., 2012). Potentially, infants’ perception of rhymes during informal language play contributes to emerging phonological awareness skills and vocabulary (Politimou et al., 2019; Krijnen et al., 2020; Franco et al., 2021). Previous studies were equivocal concerning infants’ rhyme abilities (Jusczyk et al., 1999; Hayes et al., 2000, 2009; Hahn et al., 2018), presumably due to behavioural paradigms concealing infants’ subtle processing abilities (Kooijman et al., 2008). The current study employed a passive listening EEG paradigm with 10.5-month-old Dutch infants to explore whether infants differentiate rhyming from non-rhyming pseudo-words in child songs in the absence of an explicit behavioural task. Infants’ response to the first rhymes occurring in each song (at the end of the second phrase, *rhyme sensitivity effect*) did not render significant results. For rhymes repeated throughout the song (at the end of phrase 3–10, *rhyme repetition effect*) an early negative effect for rhymes was found that approached conventional significance levels and correlated with productive vocabularies at 18 months.

For the rhyme sensitivity effect there was a numeric indication for rhyming pseudo-words to elicit a sustained positivity (Figure 2A), but the effect did not survive multiple comparison correction. The inconclusive results might be attributable to a lack of statistical power, as data from only 18 infants was available for this analysis, with each infant providing on average only seven trials per condition.

Repeated rhymes occurring at the end of phrase three to ten of the songs elicited a central negativity within the first 200 ms after pseudo-word onset, which was marginally significant when corrected for multiple comparisons. Consequently, this effect delivers tentative evidence for an implicit neural rhyme response in infants. The early negative effect for rhyming pseudo-words is similar to the early negative rhyme effect that was identified by Wagensveld et al. (2013) for single words in pre-literate 5-year-olds, but not 7-year-olds. Later negative effects for rhyming words have also been identified in 3–5 year-olds (Andersson et al., 2018). For complete words repeated in continuous speech, both negative and positive word familiarity effects have been found (see Teixidó et al., 2018). A previous study that used child songs, reported a positive word familiarity effect (Snijders et al., 2020). The opposite polarity in the current study might be attributable to rhymes occurring consistently at the end of the song phrases. The fixed rhyme position might have lessened working memory load in comparison to Snijders et al. (2020), where critical words were occurring at various phrase positions (see Snijders et al., 2020 for more background on polarity differences in the word familiarity effect). In terms of polarity, our negative rhyme effect rather adheres to other studies with recurring spoken single words and rhymes (Wagensveld et al., 2013; Andersson et al., 2018; Teixidó et al., 2018). The rhyme repetition effect observed here occurred immediately after pseudo-word onset, which is different from the word familiarity effect which usually occurs between 200 and 500 ms after word onset (Teixidó et al., 2018), but again similar to the early negative rhyme effect in 5-year-olds identified by Wagensveld et al. (2013). The latency and polarity of our effect have to be interpreted with caution, due to the effect being the result of an average over the 3rd through 10th phrase of the songs. Future studies, with more trials available per phrase of the songs, should investigate whether the effect changes (gradually) in latency and polarity upon every rhyme repetition (Kooijman et al., 2013; Nordt et al., 2016).

Infants with more negative rhyme repetition effects at 10.5 months had larger productive vocabularies at 18 months. This is the first study to report a relationship between infants’ vocabularies and their perception of repeating rhymes in songs. This finding extends previous studies, which established such relationship based on the detection of phonemes and words in fluent speech (see Cristia et al., 2014 for a review).

The functional relevance of rhyme sensitivity for infant development requires further research. Infants might experience no communicative pressure to utilise their implicit knowledge about the syllabic units of onsets and rhymes, due to their small lexicons not yet containing many rhyming words (Johnson, 2016). Rhymes in songs, however, are placed within a particularly intriguing stimulus that is highly ritualised, repetitive, rich in structural cues and progressing at a rather slow pace (Trehub et al., 1993, 1997; Trainor, 1996; Longhi, 2009; Falk and Kello, 2017). The acoustic context of language play might provide infants with a chance to recognise the syllabic structure of rhyming words, while this might be much more difficult in ordinary speech. So far, there is mounting evidence for a relationship between processing and production of spoken nursery rhymes and literacy and phonological awareness skills in pre-schoolers (see Dunst et al., 2011 for review). Based on the current study, songs and nursery rhymes might have an impact on phonological processing and vocabulary already during infancy (see also Franco et al., 2021).

The tentative relationship between implicit rhyme processing and vocabulary observed in the current study requires future replication. Only productive vocabulary, and not word comprehension, was related to early rhyme abilities. This might be due to a more reliable parental estimate of productive vocabulary. Another tentative explanation would be the use of prediction in production (van Alphen et al., 2021), which might also have impacted processing of our predictable rhyming stimuli.

The limited number of trials, electrodes and infants in our sample makes future replication and extension of the study necessary. Future research should also settle to what extent the rhyme effect reported here differs from the ERP word familiarity effect. Both effects are elicited by repeating phonological material in infants’ input and could thus stem from the same underlying auditory processing mechanism. One possible interpretation is that the rhyme effect we identify in the current study is just a word familiarity effect that appears early due to the predictability of the appearance of the rhymes at phrase ends. Solving this issue is impeded by the exclusion of anterior electrodes in the current study, the standard location of measuring the word familiarity effect. An alternative interpretation would be that the effects differ, and possibly depend on the perceived lexicality of the repeated stimuli. The ERP word familiarity effect has mainly been reported for existing words, while the rhyme negativity in pre-schoolers can be elicited by existing as well as pseudo-words (Andersson et al., 2018). Additionally, the ERP word familiarity effect arises from repetition of full words, while the rhyme negativity is based on repetition of syllable nucleus and coda only, and not other kinds of phonological overlap (Wagensveld et al., 2013). Whether infants in the current study recognise the change in syllable onsets between successive rhyming pseudo-words (e.g., *paf, taf, kaf*) or rather consider them repetitions of the same pseudo-word and ignore onset differences (e.g., *paf, paf, paf*) is another question that remains for future research (see also Ngon et al., 2013).

The current study complements and extends previous behavioural results (Hahn et al., 2018) with an ERP response for 10.5-month-old infants’ early implicit rhyme detection in a natural rhyming stimulus and a relationship of this ERP response with productive vocabulary at 18 months of age. Implicit rhyme detection in language play might contribute to the development of explicit phonological awareness abilities and vocabulary (Krijnen et al., 2020; Franco et al., 2021). The current early evidence of phonological awareness in infancy might thus reflect the origin of a key predictor of reading achievement (Wood and Terrell, 1998; Ziegler and Goswami, 2005; Melby-Lervåg et al., 2012).

## Data Availability Statement

The raw data supporting the conclusions of this article will be made available by the authors, without undue reservation.

## Ethics Statement

The studies involving human participants were reviewed and approved by the Ethical Board of the Faculty of Social Sciences, Radboud University Nijmegen, CMO2012/012. Written informed consent to participate in this study was provided by the participants’ legal guardian/next of kin.

## Author Contributions

LH and TS conceived and programmed the experiment and analysed the data. LH collected the data, made the figures, and wrote the first draft of the manuscript. All authors reviewed and edited the manuscript.

## Conflict of Interest

The authors declare that the research was conducted in the absence of any commercial or financial relationships that could be construed as a potential conflict of interest.

## Publisher’s Note

All claims expressed in this article are solely those of the authors and do not necessarily represent those of their affiliated organizations, or those of the publisher, the editors and the reviewers. Any product that may be evaluated in this article, or claim that may be made by its manufacturer, is not guaranteed or endorsed by the publisher.

## Footnotes

1
www.neurobs.com
